# IESS-FusionNet: Physiologically Inspired EEG-EMG Fusion with Linear Recurrent Attention for Infantile Epileptic Spasms Syndrome Detection

**DOI:** 10.3390/bioengineering13010057

**Published:** 2025-12-31

**Authors:** Junyuan Feng, Zhenzhen Liu, Linlin Shen, Xiaoling Luo, Yan Chen, Lin Li, Tian Zhang

**Affiliations:** 1 College of Computer Science and Software Engineering, Shenzhen University, Shenzhen 518060, China; 2300271093@email.szu.edu.cn; 2 Surgery Division, Epilepsy Center, Shenzhen Children’s Hospital, Shenzhen 518038, China; lzzqjw@163.com (Z.L.); ls19821109@163.com (L.L.); zhangtian0618@163.com (T.Z.); 3 National Engineering Laboratory of Big Data System Computing Technology, College of Computer Science and Software Engineering, Shenzhen University, Shenzhen 518060, China; 4 Department of Computer Science, University of Nottingham Ningbo China, Ningbo 315100, China; 5 Guangdong Key Laboratory of Intelligent Information Processing, Shenzhen University, Shenzhen 518060, China

**Keywords:** infantile epileptic spasms syndrome, EEG-EMG fusion, seizure detection, Mamba, linear recurrent attention, deep learning

## Abstract

Infantile Epileptic Spasms Syndrome (IESS) is a devastating epileptic encephalopathy of infancy that carries a high risk of lifelong neurodevelopmental disability. Timely diagnosis is critical, as every week of delay in effective treatment is associated with worse cognitive outcomes. Although synchronized electroencephalogram (EEG) and surface electromyography (EMG) recordings capture both the electrophysiological and motor signatures of spasms, accurate automated detection remains challenging due to the non-stationary nature of the signals and the absence of physiologically plausible inter-modal fusion in current deep learning approaches. We introduce IESS-FusionNet, an end-to-end dual-stream framework specifically designed for accurate, real-time IESS detection from simultaneous EEG and EMG. Each modality is processed by a dedicated Unimodal Encoder that hierarchically integrates Continuous Wavelet Transform, Spatio-Temporal Convolution, and Bidirectional Mamba to efficiently extract frequency-specific, spatially structured, local and long-range temporal features within a compact module. A novel Cross Time-Mixing module, built upon the linear recurrent attention of the Receptance Weighted Key Value (RWKV) architecture, subsequently performs efficient, time-decaying, bidirectional cross-modal integration that explicitly respects the causal and physiological properties of cortico-muscular coupling during spasms. Evaluated on an in-house clinical dataset of synchronized EEG-EMG recordings from infants with confirmed IESS, IESS-FusionNet achieves 89.5% accuracy, 90.7% specificity, and 88.3% sensitivity, significantly outperforming recent unimodal and multimodal baselines. Comprehensive ablation studies validate the contribution of each component, while the proposed cross-modal fusion requires approximately 60% fewer parameters than equivalent quadratic cross-attention mechanisms, making it suitable for real-time clinical deployment. IESS-FusionNet delivers an accurate, computationally efficient solution with physiologically inspired cross-modal fusion for the automated detection of infantile epileptic spasms, offering promise for future clinical applications in reducing diagnostic delay.

## 1. Introduction

Infantile epileptic spasms syndrome (IESS), also known as West syndrome, is a catastrophic, age-dependent epileptic and developmental encephalopathy with typical onset in the first year of life and peak incidence between 4 and 8 months [1,2]. Characterized by clustered epileptic spasms and frequent developmental arrest or regression [1], IESS is recognized by the International League Against Epilepsy (ILAE) as a distinct epilepsy syndrome due to its devastating long-term neurodevelopmental consequences [2,3]. Compelling evidence demonstrates that early diagnosis and prompt initiation of effective therapy are strong predictors of better cognitive outcome [4]. Notably, treatment delays exceeding three weeks after hypsarrhythmia onset are associated with significantly worse developmental trajectories [5]. Despite this narrow therapeutic window, diagnostic delays remain widespread, primarily because spasms are often subtle, easily misinterpreted by caregivers and non-specialist clinicians [6,7].

The electroencephalogram (EEG), a non-invasive neurophysiological technique that records cortical electrical activity, is fundamental to epilepsy diagnosis due to its direct measurement of neural function and high temporal resolution [8]. In IESS, ictal EEG patterns are highly heterogeneous, typically featuring high-voltage slow waves followed by abrupt attenuation (electrodecremental events), often superimposed with fast activity, spikes, or sharp waves [9]. Current clinical practice relies on expert visual review of prolonged recordings using standardized montages such as the International 10–20 system [10]. This process, however, is time-consuming, subjective, and prone to interobserver variability, directly contributing to delayed intervention [11].

Although EEG is indispensable, its interpretation is severely hampered by the intrinsically non-stationary nature of the signals and by prominent movement artifacts generated during the spasms themselves [12,13]. Synchronous surface electromyography (EMG) recorded from the proximal muscle groups offers a complementary, artifact-resistant measure of the motor manifestation of spasms and directly reflects the same pathological cortico-muscular event [14,15]. When combined, EEG and EMG therefore provide mutually reinforcing physiological signatures of IESS. Nevertheless, most existing automated detection systems either analyze these modalities independently or resort to shallow fusion strategies (e.g., simple concatenation or decision-level averaging) that fail to capture the temporally dynamic, causally directed, and decaying interplay between cortex and muscle during spasms.

Deep learning has substantially advanced automated analysis of physiological signals, yet effective and comprehensive feature extraction from non-stationary, high-dimensional recordings such as EEG remains challenging. Early methods relied on handcrafted time-frequency features paired with classical machine learning classifiers [16,17]. Although moderately successful, their performance was fundamentally limited by manually engineered features and an inability to capture hierarchical temporal dynamics. Convolutional Neural Networks (CNNs) enabled end-to-end learning of local spatial and short-range temporal patterns directly from raw or spectro-temporal inputs [18,19,20]. Standard convolutions, however, have restricted receptive fields that cannot adequately model the long-range dependencies required to represent the complete evolution of epileptic spasms. Transformers mitigated this limitation through self-attention [21,22,23,24], but their quadratic computational complexity is prohibitive for long, multichannel biosignal sequences and they lack built-in inductive biases suited to fine-grained local morphology. More recently, the Mamba architecture has emerged as an efficient alternative, achieving linear-time sequence modeling via selective state-space mechanisms while preserving strong long-range modeling capability [25]. However, its integration with multichannel time-frequency representations of biomedical signals remains largely unexplored.

In multimodal fusion, most existing approaches fail to adequately capture the clinically significant interplay between EEG and EMG. Numerous studies have confirmed performance gains from combining EEG with complementary modalities [26,27,28,29], including optimization-enhanced EEG-EMG fusion specifically applied to IESS [30]. Nevertheless, the majority still employ simple concatenation or decision-level averaging that cannot model dynamic, temporally directed interactions. While cross-attention mechanisms have been proposed to establish explicit inter-modal correspondences [31], their assumption of uniform and instantaneous correlations across entire sequences is incompatible with the causally directed, time-decaying nature of cortico-muscular communication during spasms. These mechanisms also suffer from quadratic complexity.

To overcome the limitations of existing approaches, we propose IESS-FusionNet, an end-to-end dual-stream framework tailored for automated detection of infantile epileptic spasms from synchronized EEG and EMG. Each modality is first processed by a dedicated Unimodal Encoder that hierarchically combines Continuous Wavelet Transform (CWT), Spatio-Temporal Convolution (ST-Conv), and Bidirectional Mamba (Bi-Mamba) to efficiently extract frequency-specific, spatially structured, local, and long-range temporal features within a single compact module. The resulting representations are subsequently fused through a novel Cross Time-Mixing module adapted from the Receptance Weighted Key Value (RWKV) architecture. Unlike conventional cross-attention, this module explicitly models time-decaying, causally directed, and bidirectional interactions that closely align with the physiological characteristics of cortico-muscular coupling during spasms, while maintaining linear computational complexity. By this design, IESS-FusionNet achieves a robust, physiologically plausible synthesis of EEG and EMG data, addressing both intra-modal feature extraction inadequacy and multi-modal fusion bottlenecks.

The main contributions of this work are as follows:

- We present IESS-FusionNet, an end-to-end multimodal framework that achieves accurate fusion of EEG and EMG for automated IESS detection.
- We introduce a unified Unimodal Encoder that jointly captures multi-scale frequency, spatial topology, local morphology, and global temporal dynamics of non-stationary biosignals in an efficient hierarchical design.
- We propose Cross Time-Mixing, a linear recurrent attention mechanism that enables dynamic, physiologically plausible, and bidirectional integration of EEG and EMG sequences.

The remainder of the paper is organized as follows. Section 2 describes the detailed architecture of IESS-FusionNet. Section 3 presents the clinical EEG-EMG dataset and preprocessing pipeline. Section 4 reports the experimental setup and results. Section 5 discusses the findings, and Section 6 concludes the paper.

## 2. Methods

### 2.1. Overall Architecture

**Figure 1:**
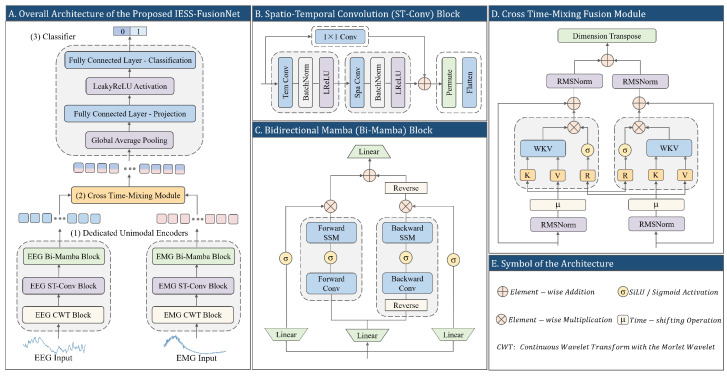
Overall architecture of the proposed IESS-FusionNet. It comprises two dedicated Unimodal Encoders, a Cross Time-Mixing module, and a Classifier.

The proposed IESS-FusionNet is an end-to-end dual-stream network designed for binary classification of epileptic spasms from synchronized EEG and EMG recordings (Figure 1). The architecture directly addresses two fundamental limitations in current automated IESS detection: (i) the inadequacy of intra-modal feature extraction from highly non-stationary, multichannel biosignals, and (ii) the lack of physiologically plausible cross-modal fusion.

IESS-FusionNet comprises three core stages: two modality-specific Unimodal Encoders, a Cross Time-Mixing fusion module, and a lightweight classifier. Each Unimodal Encoder hierarchically processes its respective signal as follows. First, a CWT block generates clinically relevant time-frequency representations. An ST-Conv block then extracts localized spatial topologies and short-range temporal patterns. Finally, a Bi-Mamba block models long-range dependencies with strictly linear complexity. The resulting high-level representations from both streams are subsequently fused by the Cross Time-Mixing module, which performs efficient, time-decaying, and bidirectional cross-modal interaction explicitly aligned with the nature of cortico-muscular transmission. The fused sequence is finally aggregated via global average pooling and classified by a two-layer MLP to produce the spasm/non-spasm decision.

### 2.2. Unimodal Encoder

#### 2.2.1. Time-Frequency Decomposition

EEG and EMG signals in IESS are highly non-stationary, exhibiting transient spikes, high-amplitude slow waves, abrupt electrodecremental events, and brief muscle contractions. Traditional Fourier-based approaches cannot adequately resolve their time-varying spectral content. We adopt the CWT with a complex Morlet mother wavelet, which offers excellent joint time-frequency localization for such pathological patterns [32].

Given a preprocessed input tensor $x\in R^{C\times T}$ (*C*: channels, *T*: sequence length), the CWT is applied independently to each channel:

(1) $$x_{\mathrm{cwt}}\left(a,b\right)=\frac{1}{\sqrt{a}}\int_{-\infty }^{\infty }x_{c}\left(t\right)\;\psi^{*}\left(\frac{t-b}{a}\right)dt,$$

where a>0 is the scale, *b* is the translation, and $\psi^{*}\left(t\right)$ is the complex conjugate of the Morlet wavelet:

$$\psi \left(t\right)=\pi^{-1/4}e^{i\omega_{0}t}e^{-t^{2}/2}.$$

Scales are chosen to correspond to physiologically relevant frequency bands [27]. For EEG: δ (1–4 Hz), θ-α (4–13 Hz), β (13–30 Hz), and γ (30–70 Hz). For EMG: 5–70 Hz, 70–150 Hz, 150–250 Hz, and 250–500 Hz. The magnitude of the complex coefficients forms scalograms, resulting in the output tensor $x_{\mathrm{cwt}}\in R^{F\times C\times T}$, where F=4 denotes the number of selected frequency bands. This representation effectively preserves the multi-scale temporal dynamics critical for subsequent spatial and temporal processing.

#### 2.2.2. Spatio-Temporal Feature Extraction

Following time-frequency decomposition, the resulting scalograms reveal rich spatio-temporal patterns specific to each modality. In EEG, these encode dynamic cortical connectivity across electrodes, which is critical to capture IESS-related patterns [33]. In EMG, they reflect coordinated activation of muscle groups over time [14]. To efficiently extract these localized features, we employ a depthwise ST-Conv block comprising separable temporal and spatial convolutions followed by a residual connection.

Given the CWT output $x_{\mathrm{cwt}}\in R^{F\times C\times T}$, a temporal convolution with kernel size $\left(1,K_{t}\right)$ and $K_{t}=25$ is first applied along the time axis, followed by batch normalization and LeakyReLU activation. This yields an intermediate representation $z^{\left(1\right)}\in R^{F^{\prime }\times C\times T}$, where $F^{\prime }=16$ is the intermediate feature channels:

(2) $$z^{\left(1\right)}=\sigma_{\mathrm{LeakyReLU}}\left(\mathrm{BN}\left({\mathrm{Conv}}_{1\times K_{t}}\left(x_{\mathrm{cwt}}\right)\right)\right).$$

A subsequent spatial convolution with kernel size $K_{s}\times 1$ ($K_{s}=13$ for EEG, $K_{s}=3$ for EMG) models electrode-wise topological correlations, producing the main feature map $z^{\left(2\right)}\in R^{F^{\prime \prime }\times C\times T}$, where $F^{\prime \prime }=4$ is the final number of feature channels:

(3) $$z^{\left(2\right)}=\sigma_{\mathrm{LeakyReLU}}\left(\mathrm{BN}({\mathrm{Conv}}_{K_{s}\times 1}\left(z^{\left(1\right)}\right))\right).$$

A residual connection preserves frequency-specific information and stabilizes training:

(4) $$z_{\mathrm{out}}=z^{\left(2\right)}+{\mathrm{Conv}}_{1\times 1}\left(x_{\mathrm{cwt}}\right).$$

The resulting tensor $z_{\mathrm{out}}\in R^{F^{\prime \prime }\times C\times T}$ is transposed to $R^{T\times F^{\prime \prime }\times C}$ and flattened along the frequency and channel dimensions to form the sequence $x_{\mathrm{stc}}\in R^{T\times D}$, where $D=F^{\prime \prime }\times C$. This compact sequence, enriched with localized spatio-temporal structure, serves as input to the subsequent long-range modeling stage.

#### 2.2.3. Global Sequence Modeling

The ST-Conv block effectively captures local spatio-temporal morphology but cannot integrate long-range contextual information. Although Transformers excel at global modeling through self-attention [21,22,23,24], their quadratic complexity is prohibitive for high-resolution biosignals. The Mamba selective state-space model overcomes this limitation by offering linear-time sequence processing while preserving strong long-range dependency modeling [34]. Since epileptic spasms manifest as stereotyped temporal sequences (e.g., an abrupt cortical spike followed by electrodecremental attenuation and subsequent muscle contraction), bidirectional context is essential for robust recognition. We therefore propose a Bidirectional Mamba (Bi-Mamba) block.

Given the output of the ST-Conv block $x_{\mathrm{stc}}\in R^{T\times D}$, a linear projection expands the feature dimension to 2E (E=2D), after which the tensor is split channel-wise into forward and backward streams:

(5) $$x_{\mathrm{fwd}},x_{\mathrm{bwd}}=\mathrm{split}(\mathrm{Linear}(x_{\mathrm{stc}}))\in R^{T\times E}.$$

Each stream is processed by a standard Mamba block. For the forward pass, a causal 1-D convolution (kernel size of 4) followed by SiLU activation generates time-varying SSM parameters $\Delta_{t}\in R^{E}$, $B_{t}\in R^{E\times N}$, and $C_{t}\in R^{E\times N}$ (N=32):

(6) $$x_{\mathrm{fwd}}^{\prime }=\sigma_{\mathrm{SiLU}}\left(Conv1D(x_{\mathrm{fwd}})\right).$$

Discretization follows the standard Mamba formulation with fixed diagonal $A\in R^{N\times N}$:

(7) $$\begin{array}{cc} {\bar{A}}_{t} & =\exp(\Delta_{t}A), \end{array}$$

(8) $$\begin{array}{cc} {\bar{B}}_{t} & =\left(\exp\left(\Delta_{t}A\right)-I\right)A^{-1}B_{t}. \end{array}$$

The recurrent state and output at step *t* are

(9) $$\begin{array}{cc} h_{t} & ={\bar{A}}_{t}h_{t-1}+{\bar{B}}_{t}x_{\mathrm{fwd},t}^{\prime }, \end{array}$$

(10) $$\begin{array}{cc} y_{\mathrm{fwd},t} & =C_{t}h_{t}+D_{t}x_{\mathrm{fwd},t}^{\prime }, \end{array}$$

where $D_{t}$ is a learnable skip connection. The backward stream processes the time-reversed sequence identically, with its output subsequently reversed.

A parallel SiLU-activated gating path modulates each direction:

(11) $$y_{\mathrm{fwd},t}^{\prime }=y_{\mathrm{fwd},t}⊙\sigma_{\mathrm{SiLU}}{\left(\mathrm{Linear}(x_{\mathrm{fwd}})\right)}_{t}.$$

The gated forward and reversed backward sequences are summed and projected back to dimension *D*:

(12) $$x_{\mathrm{enc}}=\mathrm{Linear}\left(y_{\mathrm{fwd}}^{\prime }+\mathrm{Reverse}\left(y_{\mathrm{bwd}}^{\prime }\right)\right)\in R^{T\times D}.$$

This produces the final unimodal representation that integrates bidirectional long-range dependencies with strictly linear complexity.

### 2.3. Cross-Modal Fusion

Conventional multimodal fusion strategies (e.g., concatenation or late averaging) fail to capture the dynamic, causally directed interplay between EEG and EMG during spasms. Cross-attention mechanisms, despite enabling explicit inter-modal alignment, incur quadratic complexity and assume uniform instantaneous correlations—properties incompatible with the time-decaying nature of cortico-muscular coupling in IESS. We propose Cross Time-Mixing, a linear recurrent attention module adapted from the RWKV time-mixing paradigm [35]. This module achieves efficient bidirectional cross-modal interaction with linear computational complexity while incorporating time-decaying recurrence that is physiologically inspired by the temporal attenuation inherent in neuro-muscular signal propagation during spasms.

Let $x_{\mathrm{eeg}},x_{\mathrm{emg}}\in R^{T\times D}$ denote the encoded unimodal sequences. Each is first normalized using RMSNorm [36]:

(13) $$\begin{array}{cc} {\tilde{x}}_{\mathrm{eeg}} & =\mathrm{RMSNorm}(x_{\mathrm{eeg}}), \end{array}$$

(14) $$\begin{array}{cc} {\tilde{x}}_{\mathrm{emg}} & =\mathrm{RMSNorm}(x_{\mathrm{emg}}). \end{array}$$

Fusion is performed symmetrically in both directions. For the EEG stream conditioned on EMG, a receptance vector blends current and previous states:

(15) $$r_{t}^{\mathrm{eeg}}=W_{r}\left(\mu_{r}⊙{\tilde{x}}_{\mathrm{eeg},t}+\left(1-\mu_{r}\right)⊙{\tilde{x}}_{\mathrm{eeg},t-1}\right),$$

where $W_{r},\mu_{r}\in R^{D\times D}$ are learnable. Key and value vectors are derived from the EMG stream analogously:

(16) $$\begin{array}{cc} k_{t}^{\mathrm{emg}} & =W_{k}\left(\mu_{k}⊙{\tilde{x}}_{\mathrm{emg},t}+\left(1-\mu_{k}\right)⊙{\tilde{x}}_{\mathrm{emg},t-1}\right), \end{array}$$

(17) $$\begin{array}{cc} v_{t}^{\mathrm{emg}} & =W_{v}\left(\mu_{v}⊙{\tilde{x}}_{\mathrm{emg},t}+\left(1-\mu_{v}\right)⊙{\tilde{x}}_{\mathrm{emg},t-1}\right). \end{array}$$

The core channel-wise weighted key-value (WKV) recurrence uses a learnable time-decay $\omega \in R^{D}$:

(18) $$\begin{array}{cc} s_{t}^{\mathrm{emg}} & =\lambda ⊙s_{t-1}^{\mathrm{emg}}+e^{k_{t}^{\mathrm{emg}}}, \end{array}$$

(19) $$\begin{array}{cc} p_{t}^{\mathrm{emg}} & =\lambda ⊙p_{t-1}^{\mathrm{emg}}+e^{k_{t}^{\mathrm{emg}}}⊙v_{t}^{\mathrm{emg}}, \end{array}$$

where $\lambda =e^{-\omega }$. The fused context for EEG at step *t* is

(20) $${\mathrm{wkv}}_{t}^{\mathrm{eeg}\leftarrow \mathrm{emg}}=\frac{p_{t}^{\mathrm{emg}}+e^{u+k_{t}^{\mathrm{emg}}}⊙v_{t}^{\mathrm{emg}}}{s_{t}^{\mathrm{emg}}+e^{u+k_{t}^{\mathrm{emg}}}},$$

with trainable offset $u\in R^{D}$. The output is gated and projected:

(21) $$o_{t}^{\mathrm{eeg}}=W_{o}\left(\sigma \left(r_{t}^{\mathrm{eeg}}\right)⊙{\mathrm{wkv}}_{t}^{\mathrm{eeg}\leftarrow \mathrm{emg}}\right).$$

The symmetric EMG-conditioned stream yields $o_{t}^{\mathrm{emg}}$. Final fusion is obtained via residual addition and normalization:

(22) $$x_{\mathrm{fused}}=\mathrm{RMSNorm}\left(x_{\mathrm{eeg}}+o_{t}^{\mathrm{eeg}}\right)+\mathrm{RMSNorm}\left(x_{\mathrm{emg}}+o_{t}^{\mathrm{emg}}\right).$$

This produces the fused representation $x_{\mathrm{fused}}\in R^{T\times D}$ with physiologically inspired cross-modal synthesis at linear computational complexity.

### 2.4. Classifier

The fused sequence $x_{\mathrm{fused}}$ is temporally aggregated via global average pooling:

(23) $$x_{\mathrm{pool}}=\frac{1}{T}\sum_{t=1}^{T}x_{\mathrm{fused},t}\in R^{D}.$$

A two-layer MLP generates the final logit:

(24) $$\begin{array}{cc} h & =\mathrm{LeakyReLU}\left(W_{1}x_{\mathrm{pool}}+b_{1}\right)\in R^{D/2}, \end{array}$$

(25) $$\begin{array}{cc} z & =W_{2}h+b_{2}\in R^{2}, \end{array}$$

followed by binary cross-entropy loss during training to differentiate IESS events from non-IESS events.

## 3. Clinical Dataset

### 3.1. Data Source

The clinical dataset was acquired at Shenzhen Children’s Hospital using synchronized video-EEG-EMG monitoring. This retrospective study was approved by the hospital’s Ethics Committee (No. 202305802) and conducted in accordance with the Declaration of Helsinki. Written informed consent was obtained from the parents or legal guardians of all participants.

EEG was recorded with a 25-channel montage using the extended International 10–20 system (Fp1, Fp2, F7, F3, Fz, F4, F8, T3, C3, Cz, C4, T4, T5, P3, Pz, P4, T6, O1, O2) with additional electrodes (AFz, Oz, C5, C6, S1, S2). Surface EMG was simultaneously collected from the deltoid and quadriceps muscles bilaterally (four channels in total). Both modalities were sampled at 1024 Hz. The dataset comprises 129 multi-session recordings from 10 pediatric patients with confirmed IESS. Spasm events were annotated at 0.1-s resolution by three senior pediatric neurologists based on clinical observations, EEG-EMG analysis, and consensus among the three raters. This rigorous procedure yielded a total of 1941 high-density, precisely annotated spasm events. Patient demographics and dataset characteristics are summarized in Table 1 and Table 2, respectively.

### 3.2. Data Preprocessing

The EEG signals were bandpass-filtered (0.3–70 Hz), notch-filtered at 50 Hz, and re-referenced to the average. The EMG signals were bandpass-filtered between 5 and 500 Hz. Both modalities were normalized to zero mean and unit variance to facilitate model convergence.

To construct training samples, 1-s analysis windows were used. Ictal windows were densely sampled with a 0.2-s stride through all 1941 annotated spasm events, providing rich temporal coverage of each spasm. Non-ictal windows were sampled without overlap from interictal periods and subsampled to achieve class balance. The dataset was split by subject into training, validation, and test sets in an 8:1:1 ratio.

## 4. Experimental Results

### 4.1. Implementation Details

Model development was performed in Python 3.8 using the PyTorch 1.9.0 framework, with acceleration provided by an NVIDIA V100 GPU (32 GB of VRAM). Training utilized Adam optimizer with an initial learning rate of $1\times 10^{-4}$, a batch size of 128, and a maximum of 100 epochs. Early stopping was applied, terminating training if the validation loss did not improve for 10 consecutive epochs. The model was restored to the state with the lowest validation loss.

### 4.2. Evaluation Metrics

In this study, three evaluation metrics, including accuracy, specificity, and sensitivity, are adopted to evaluate the classification performance of our IESS-FusionNet, abbreviated as acc, spe, and sen, respectively. These metrics are defined as follows:

(26) $$\begin{array}{cc} \mathrm{acc} & =\frac{\mathrm{TP}+\mathrm{TN}}{\mathrm{TP}+\mathrm{TN}+\mathrm{FP}+\mathrm{FN}}, \end{array}$$

(27) $$\begin{array}{cc} \mathrm{spe} & =\frac{\mathrm{TN}}{\mathrm{TN}+\mathrm{FP}}, \end{array}$$

(28) $$\begin{array}{cc} \mathrm{sen} & =\frac{\mathrm{TP}}{\mathrm{TP}+\mathrm{FN}}. \end{array}$$

Here, TP, TN, FP and FN denote true positive, true negative, false positive, and false negative, respectively. The results are expressed as mean ± standard deviation for five independent experiments.

### 4.3. Comparative Performance

To validate the efficacy of IESS-FusionNet, we conducted a comprehensive comparative analysis against state-of-the-art (SOTA) methods, fusion strategies, and modal configurations. The results are summarized in Table 3.

**Figure 2:**
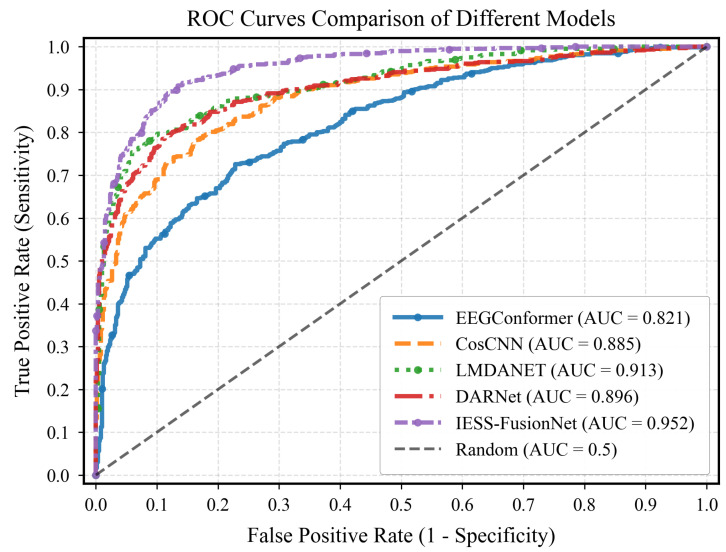
ROC curves and their corresponding AUCs for our model and SOTA methods.

**Comparison with SOTA Methods.** IESS-FusionNet outperforms SOTA methods, achieving an accuracy of 89.5%, specificity of 90.7%, and sensitivity of 88.3%. Compared to the baselines LMDANet [22] and DARNet [23], it improves accuracy by 5.2% and 8.3%, and sensitivity by 11.1% and 12.6%, respectively. This superior performance underscores the effectiveness of our comprehensive unimodal feature extraction and sophisticated multimodal fusion strategy in capturing the intricate dynamics of IESS, which single-modality EEG models often miss. Figure 2 shows the receiver operating characteristic (ROC) curves and the corresponding AUC values for IESS-FusionNet and other tested methods. The proposed model’s ROC curve lies above the others, near the top left corner, with an AUC of 0.952 that surpasses the leading SOTA.

**Comparison of Fusion Strategies.** The results from the fusion strategy comparison highlight the critical role of our Cross Time-Mixing module. It achieves a sensitivity of 88.3%, surpassing basic fusion techniques such as concatenation (81.8%) and averaging (85.8%). More importantly, our method also outperforms widely used cross-attention (86.3%). This demonstrates that the linear recurrent attention fusion is more adept at modeling the dynamic and synergistic interactions between EEG and EMG.

**Comparison of Modalities.** Multi-modal fusion (EEG + EMG) yields a 2.6% higher accuracy than EEG-only (86.9%) and a 27.1% improvement over EMG-only (62.4%). The poor performance of EMG-only reflects its limited ability to capture primary neurological events, underscoring the necessity of combining complementary EEG and EMG modalities for robust IESS diagnosis.

### 4.4. Ablation Study on Unimodal Encoder Components

To quantify the individual contribution of each component within the proposed Unimodal Encoder, we conducted an ablation study. The results presented in Table 4 demonstrate the necessity of each meticulously designed component.

The full encoder achieves optimal performance. Removing CWT causes the largest performance drop (7.7% in accuracy, 14.0% in sensitivity), underscoring its role in capturing non-stationary time-frequency dynamics. Omitting ST-Conv reduces accuracy by 3.9% and sensitivity by 5.3%, indicating its importance for local spatio-temporal feature extraction. Excluding Bi-Mamba decreases accuracy by 1.8% and sensitivity by 4.7%, confirming its contribution to global sequence modeling. These results validate the necessity of each component in addressing intra-modal feature extraction challenges.

**Figure 3:**
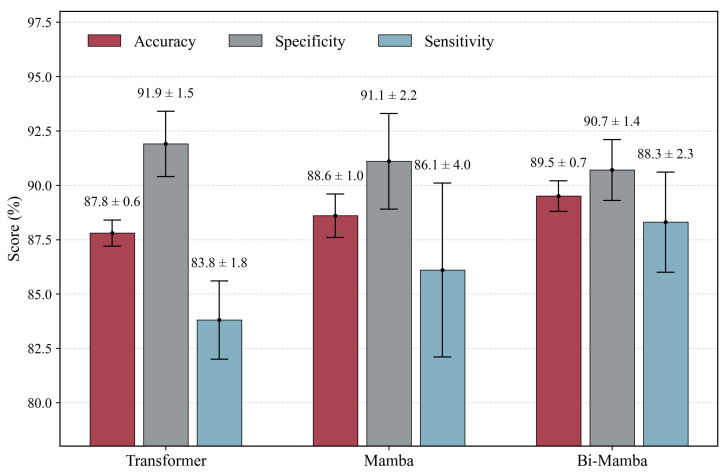
Comparison of global sequence modeling architectures.

A comparison of global sequence modeling architectures (Transformer, Mamba, Bi-Mamba) is shown in Figure 3. Bi-Mamba outperforms both Transformer and unidirectional Mamba, achieving an improvement of 2.2% in sensitivity over Mamba. This highlights the advantage of bidirectional modeling in capturing forward and backward temporal dependencies in EEG-EMG sequences, critical for IESS event detection.

### 4.5. Computational Efficiency Analysis

To assess practical feasibility, the computational efficiency of Bi-Mamba and Cross Time-Mixing was compared against Transformer and Cross-Attention, respectively, in terms of parameters (Params) and floating-point operations (FLOPs) per forward pass. The results are shown in Table 5.

**Global Sequence Modeling Efficiency.** Bi-Mamba significantly reduces computational overhead compared to Transformer, requiring 0.25 M parameters and 0.22 GFLOPs versus 0.78 M parameters and 0.80 GFLOPs. While unidirectional Mamba is more efficient (0.17M parameters, 0.08 GFLOPs), Bi-Mamba’s marginal increase in cost yields superior performance (Figure 3), offering a balanced trade-off for clinical applications.

**Cross-Modal Fusion Efficiency.** The Cross Time-Mixing module requires 0.23 M parameters and 0.75 GFLOPs, achieving approximately a 60% reduction in parameters and 48% in FLOPs compared to Cross-Attention (0.58 M parameters and 1.43 GFLOPs). This efficiency enables scalable processing of long EEG-EMG sequences, which is essential for potential real-time IESS detection in clinical settings.

### 4.6. Feature Visualization

To provide intuitive insight into the progressive refinement of discriminative representations within IESS-FusionNet, we present topographic maps of learned spatial patterns and t-Distributed Stochastic Neighbor Embedding (t-SNE) projections of high-dimensional features at key stages of the pipeline.

**Figure 4:**
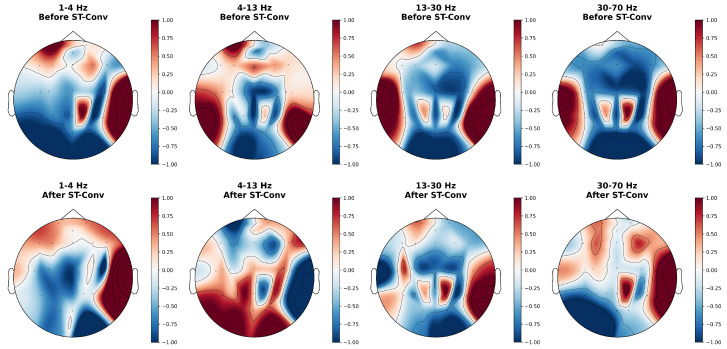
EEG topographic maps before and after ST-Conv block across different frequency bands.

As illustrated in Figure 4, ST-Conv demonstrates distinct optimization effects on EEG topomaps across different frequency bands. In the low-frequency δ band (1–4 Hz), the block’s function is primarily spatial smoothing and denoising. Isolated, noise-like hotspots in the frontal lobe are eliminated, resulting in a diffuse, bilateral uniform activation. Concurrently, isolated activity in the parietal lobe is smoothed into background inhibition, while key signals in the right temporal lobe are robustly preserved. In the mid-frequency θ and α bands (4–13 Hz), ST-Conv achieves significant energy redistribution, effectively suppressing high-amplitude artifacts in the prefrontal region while enhancing coherent activation in the occipital area. In the high-frequency β and γ bands (13–70 Hz), the block performs lateralization adjustments that disrupt false bilateral symmetry caused by factors such as EMG interference. Redundant signals in the left temporal lobe are substantially attenuated, whereas strong activation in the right temporal lobe is retained, thereby underscoring right-hemispheric dominance.

**Figure 5:**
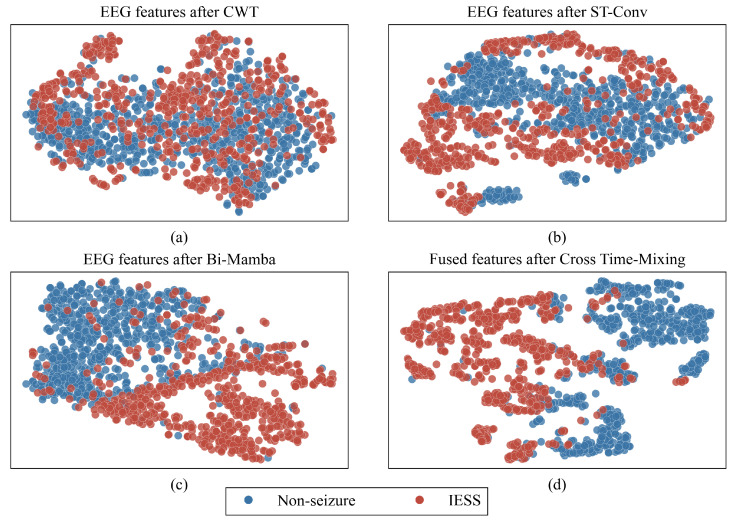
t-SNE visualization of feature evolution: (**a**) post-CWT, (**b**) post-ST-Conv, (**c**) post-Bi-Mamba, and (**d**) final fused representation after Cross Time-Mixing.

Figure 5 further elucidates the hierarchical construction of discriminative features via t-SNE visualization of the evolving latent space. Subfigure (a) reveals considerable overlap between IESS and non-IESS samples when only raw CWT scalograms are used. After ST-Conv (b), local spatio-temporal refinement yields noticeably more compact intra-class clusters and initial inter-class separation. The subsequent Bi-Mamba stage (c) leverages bidirectional long-range modeling to sharpen class boundaries further, producing highly discriminative unimodal representations. Finally, the Cross Time-Mixing module (d) integrates complementary EEG and EMG information, achieving optimal separability in the fused space. This progressive, monotonic increase in class discriminability quantitatively corroborates the synergistic contribution of each component and underscores the neurophysiological plausibility of the proposed cross-modal fusion.

## 5. Discussion

The superior performance of Cross Time-Mixing over conventional fusion strategies underscores the benefit of modeling temporal dynamics and directionality in cortico-muscular interactions. Cross-attention computes a global affinity matrix that assumes uniform correlation across the entire sequence, leading to quadratic complexity and redundant computation for synchronized physiological signals. In contrast, the proposed RWKV-inspired recurrent formulation incorporates explicit time-decaying weights, which better capture the directional and attenuating characteristics of signal propagation during spasms. This design achieves a sensitivity of 88.3%, compared to 86.3% for quadratic cross-attention (Table 3), while requiring approximately 60% fewer parameters (Table 5). The consistent improvement suggests that emphasizing temporal directionality and decay helps the model more effectively distinguish true spasms from motion artifacts, which typically lack such structured inter-modal synchronization.

Ablation studies confirm the necessity of hierarchical feature extraction within each modality. Removing the CWT or ST-Conv block produces marked performance declines (Table 4), underscoring the challenge posed by the non-stationary, low signal-to-noise characteristics of clinical IESS recordings. As evidenced in the topographic visualizations (Figure 4), the ST-Conv block effectively acts as a learnable filter. In the high-frequency β and γ bands, the model suppresses bilateral background noise and highlights focal activation in the temporal regions. This aligns with clinical observations that, although spasms are generalized, they often exhibit focal initiation or asymmetry corresponding to underlying structural abnormalities [37]. Furthermore, the Bi-Mamba component effectively captures long-range temporal dependencies in spasm events. It allows the model to better understand the sequential nature of spasms, distinguishing key phases such as pre-ictal buildup, ictal spikes, and post-ictal attenuation. As demonstrated in Figure 5, Bi-Mamba renders the separation between IESS and non-IESS clusters remarkably distinct through bidirectional global modeling, enabling the extraction of highly discriminative features. The performance degradation observed when removing any of these components indicates that a hierarchical combination, transitioning from time-frequency decomposition to localized spatio-temporal filtering and finally to long-range sequence modeling, is critical for achieving robust representation of these complex events on challenging, real-world clinical data. In addition, this hierarchical design contributes to computational efficiency. Within the dual-stream Unimodal Encoders, the CWT block contains no learnable parameters, and the ST-Conv block contributes approximately 4.4 K parameters in total. The majority of the parameters stem from the Bi-Mamba block (0.25 M, Table 5). This results in a relatively low overall parameter count that supports comprehensive extraction of multi-scale frequency, local spatio-temporal, and long-range temporal features, particularly compared to Transformer-based encoders.

Despite the promising performance of IESS-FusionNet, which achieved significantly higher sensitivity compared to recent baselines [20,21,22,23] on the independent test set of the same clinical dataset, the current study has limitations that warrant discussion. Primarily, the sample size is relatively limited (129 recordings from 10 patients), due to the rarity of IESS, with an estimated incidence of approximately 2 per 10,000 live births [3]. The small number of subjects raises concerns about subject-level generalization, although a simple subject-independent random split was used for training, validation, and testing. Although the dataset includes multiple sessions per patient and was meticulously annotated by experienced pediatric neurologists, this constrained cohort may not fully represent the broader inter-patient variability encountered in larger, multi-center populations, potentially affecting the model’s generalizability to diverse recording equipment, patient demographics, etiologies, and clinical settings. Future studies should prioritize validation on independent, larger-scale datasets to more rigorously assess robustness and generalization performance. Additionally, employing strategies such as leave-one-patient-out cross-validation or domain adaptation techniques could further mitigate overfitting risks in small cohorts.

Beyond expanding the dataset through ongoing multi-center collaborations, we plan to integrate EEG-EMG representations generated by IESS-FusionNet with prompt-driven embeddings from compact open-source large language models (e.g., Qwen-2.5-7B). By fine-tuning the selected LLM on multimodal inputs, we aim to enable simultaneous classification and generation of brief, clinically interpretable reports (e.g., “high-voltage slow waves followed by electrodecremental attenuation with synchronous proximal muscle contraction”).

## 6. Conclusions

In this paper, we proposed IESS-FusionNet, a multimodal deep learning framework designed for the accurate detection of IESS. By combining dedicated Unimodal Encoders with an efficient Cross Time-Mixing fusion mechanism, the model effectively captures frequency-specific patterns, spatio-temporal structure, long-range dependencies, and the physiologically plausible time-decaying cortico-muscular coupling of synchronized EEG and EMG signals. Experimental results on the clinical dataset demonstrate that the proposed model outperforms recent unimodal and multimodal baselines while maintaining linear computational complexity and requiring substantially fewer parameters. These advantages make IESS-FusionNet promising for real-time clinical deployment, with the potential to reduce diagnostic delay and enable earlier therapeutic intervention in this severe epileptic encephalopathy of infancy.

## Figures and Tables

**Table 1:** Patient demographics and clinical information.

ID	Gender	Age	Seizure Count	Seizure Time (s)
a	Female	1y6m	156	205.1
c	Male	10m	62	148.3
d	Female	11m	86	87.1
f	Male	10m	357	488.4
g	Male	2y4m	29	25.5
h	Female	11m	52	60.1
i	Female	1y5m	468	580.7
l	Female	5m	338	297.6
m	Male	3y	139	139.8
n	Male	4y	254	449.1

**Table 2:** Summary of the clinical dataset characteristics.

Attribute	Value
Total Seizure Events	1941
Total Seizure Duration	2481.7 s
Shortest/Longest Duration	0.4 s/9.2 s
Number of Recordings	129 EEG-EMG recordings
Total Recording Duration	630 min
Acquisition Device	Compumedics Grael
Sampling Rate	1024 Hz
Electrode Type	Disk electrodes
Number of Electrodes	25 (EEG), 4 (EMG)
Placement System	International 10–20 system
Recording Environment	Hospital epilepsy monitoring ward

**Table 3:** Comparative performance of IESS-FusionNet against baseline SOTA methods, different fusion strategies, and modality configurations.

Method	Acc (%)	Spe (%)	Sen (%)
SOTA Methods Comparison			
EEGConformer [21]	77.5 ± 2.1	82.1 ± 1.6	72.9 ± 4.9
CosCNN [20]	81.5 ± 0.8	88.7 ± 1.8	74.4 ± 2.2
LMDANET [22]	84.3 ± 1.0	**91.4 ± 1.1**	77.2 ± 1.3
DARNet [23]	81.2 ± 0.9	86.7 ± 2.3	75.7 ± 2.6
IESS-FusionNet	**89.5 ± 0.7**	90.7 ± 1.4	**88.3 ± 2.3**
Fusion Strategy Comparison			
Concatenation	87.3 ± 1.2	**92.7 ± 0.7**	81.8 ± 3.1
Averaging	88.2 ± 0.8	90.6 ± 2.1	85.8 ± 1.9
Cross-Attention	88.1 ± 1.2	89.9 ± 0.9	86.3 ± 2.2
Cross Time-Mixing	**89.5 ± 0.7**	90.7 ± 1.4	**88.3 ± 2.3**
Modality Comparison			
EEG-only	86.9 ± 1.6	88.3 ± 3.5	85.6 ± 1.5
EMG-only	62.4 ± 0.7	76.7 ± 5.8	48.1 ± 7.0
EEG + EMG	**89.5 ± 0.7**	**90.7 ± 1.4**	**88.3 ± 2.3**

**Table 4:** Ablation study on Unimodal Encoder components.

Configuration	Acc (%)	Spe (%)	Sen (%)
Full Encoder	**89.5 ± 0.7**	90.7 ± 1.4	**88.3 ± 2.3**
w/o CWT	81.8 ± 1.7	89.3 ± 1.7	74.3 ± 2.3
w/o ST-Conv	85.6 ± 1.2	88.2 ± 2.5	83.0 ± 0.7
w/o Bi-Mamba	87.7 ± 2.2	**91.9 ± 1.0**	83.6 ± 4.4

**Table 5:** Computational efficiency comparison of global sequence modeling and Fusion Methods.

Component	Params (M)	FLOPs (G)
Global Sequence Modeling		
Transformer	0.78	0.80
Mamba	0.17	0.08
Bi-Mamba	0.25	0.22
Cross-Modal Fusion		
Cross-Attention	0.58	1.43
Cross Time-Mixing	0.23	0.75

## Data Availability

The data that support the findings of this study are proprietary to Shenzhen Children’s Hospital and are not openly available due to reasons of sensitivity. They are available from the corresponding author (ccflysz@126.com) upon reasonable request.

## Abbreviations

The following abbreviations are used in this manuscript: IESS: Infantile Epileptic Spasms Syndrome; ILAE: International League Against Epilepsy; EEG: Electroencephalogram; EMG: Electromyography; CWT: ContinuousWavelet Transform; ST-Conv: Spatio-Temporal Convolution; Bi-Mamba: Bidirectional Mamba; RWKV: ReceptanceWeighted Key Value; ACC: Accuracy; SPE: Specificity; SEN: Sensitivity; ROC: Receiver Operating Characteristic Curve; AUC: Area Under the ROC Curve; SOTA: State-of-the-Art; T-SNE: t-Distributed Stochastic Neighbor Embedding; FLOPs: Floating Point Operations; LLMs: Large Language Models.

## References

[1] Pavone P., Striano P., Falsaperla R., Pavone L., Ruggieri M. (2014). Infantile spasms syndrome, West syndrome and related phenotypes: What we know in 2013. Brain Dev..

[2] Specchio N., Wirrell E.C., Scheffer I.E., Nabbout R., Riney K., Samia P., Guerreiro M., Gwer S., Zuberi S.M., Wilmshurst J.M. (2022). International League Against Epilepsy classification and definition of epilepsy syndromes with onset in childhood: Position paper by the ILAE Task Force on Nosology and Definitions. Epilepsia.

[3] Romero Milà B., Remakanthakurup Sindhu K., Mytinger J.R., Shrey D.W., Lopour B.A. (2022). EEG biomarkers for the diagnosis and treatment of infantile spasms. Front. Neurol..

[4] Riikonen R.S. (2010). Favourable prognostic factors with infantile spasms. Eur. J. Paediatr. Neurol..

[5] Primec Z.R., Stare J., Neubauer D. (2006). The risk of lower mental outcome in infantile spasms increases after three weeks of hypsarrhythmia duration. Epilepsia.

[6] Napuri S., Le Gall E., Dulac O., Chaperon J., Riou F. (2010). Factors associated with treatment lag in infantile spasms. Dev. Med. Child Neurol..

[7] Hussain S.A., Lay J., Cheng E., Weng J., Sankar R., Baca C.B. (2017). Recognition of infantile spasms is often delayed: The ASSIST study. J. Pediatr..

[8] Teplan M. (2002). Fundamentals of EEG measurement. Meas. Sci. Rev..

[9] Watanabe K., Negoro T., Aso K., Matsumoto A. (1993). Reappraisal of interictal electroencephalograms in infantile spasms. Epilepsia.

[10] Oostenveld R., Praamstra P. (2001). The five percent electrode system for high-resolution EEG and ERP measurements. Clin. Neurophysiol..

[11] Cao J., Chen Y., Zheng R., Cui X., Jiang T., Gao F. (2023). DSMN-ESS: Dual-stream multitask network for epilepsy syndrome classification and seizure detection. IEEE Trans. Instrum. Meas..

[12] Guo Y., Jiang X., Tao L., Meng L., Dai C., Long X., Wan F., Zhang Y., Van Dijk J., Aarts R.M. (2022). Epileptic seizure detection by cascading isolation forest-based anomaly screening and EasyEnsemble. IEEE Trans. Neural Syst. Rehabil. Eng..

[13] Zheng R., Feng Y., Wang T., Cao J., Wu D., Jiang T., Gao F. (2022). Scalp EEG functional connection and brain network in infants with West syndrome. Neural Netw..

[14] Zhang F., Li P., Hou Z.G., Lu Z., Chen Y., Li Q., Tan M. (2012). sEMG-based continuous estimation of joint angles of human legs by using BP neural network. Neurocomputing.

[15] Xi X., Sun Z., Hua X., Yuan C., Zhao Y.B., Miran S.M., Luo Z., Lü Z. (2021). Construction and analysis of cortical–muscular functional network based on EEG-EMG coherence using wavelet coherence. Neurocomputing.

[16] Siddiqui M.K., Morales-Menendez R., Huang X., Hussain N. (2020). A review of epileptic seizure detection using machine learning classifiers. Brain Inform..

[17] Shen M., Wen P., Song B., Li Y. (2022). An EEG based real-time epilepsy seizure detection approach using discrete wavelet transform and machine learning methods. Biomed. Signal Process. Control.

[18] Wei B., Zhao X., Shi L., Xu L., Liu T., Zhang J. (2021). A deep learning framework with multi-perspective fusion for interictal epileptiform discharges detection in scalp electroencephalogram. J. Neural Eng..

[19] Pan Y., Zhou X., Dong F., Wu J., Xu Y., Zheng S. (2022). Epileptic seizure detection with hybrid time-frequency EEG input: A deep learning approach. Comput. Math. Methods Med..

[20] Liu G., Tian L., Wen Y., Yu W., Zhou W. (2024). Cosine convolutional neural network and its application for seizure detection. Neural Netw..

[21] Song Y., Zheng Q., Liu B., Gao X. (2022). EEG conformer: Convolutional transformer for EEG decoding and visualization. IEEE Trans. Neural Syst. Rehabil. Eng..

[22] Miao Z., Zhao M., Zhang X., Ming D. (2023). LMDA-Net: A lightweight multi-dimensional attention network for general EEG-based brain-computer interfaces and interpretability. NeuroImage.

[23] Yan S., Fan C., Zhang H., Yang X., Tao J., Lv Z. (2024). Darnet: Dual attention refinement network with spatiotemporal construction for auditory attention detection. Adv. Neural Inf. Process. Syst..

[24] Zhu R., Pan W.X., Liu J.X., Shang J.L. (2024). Epileptic seizure prediction via multidimensional transformer and recurrent neural network fusion. J. Transl. Med..

[25] Lu G., Peng J., Huang B., Gao C., Stefanov T., Hao Y., Chen Q. SlimSeiz: Efficient Channel-Adaptive Seizure Prediction Using a Mamba-Enhanced Network. Proceedings of the 2025 IEEE International Symposium on Circuits and Systems (ISCAS).

[26] Verma G.K., Tiwary U.S. (2014). Multimodal fusion framework: A multiresolution approach for emotion classification and recognition from physiological signals. NeuroImage.

[27] Al-Quraishi M.S., Elamvazuthi I., Tang T.B., Al-Qurishi M., Parasuraman S., Borboni A. (2021). Multimodal fusion approach based on EEG and EMG signals for lower limb movement recognition. IEEE Sens. J..

[28] Kim S., Shin D.Y., Kim T., Lee S., Hyun J.K., Park S.M. (2022). Enhanced recognition of amputated wrist and hand movements by deep learning method using multimodal fusion of electromyography and electroencephalography. Sensors.

[29] Bhatlawande S., Shilaskar S., Pramanik S., Sole S. (2024). Multimodal emotion recognition based on the fusion of vision, EEG, ECG, and EMG signals. Int. J. Electr. Comput. Eng. Syst..

[30] Wu D., Zhang W., Jiang L., Zhang L., Vidal P.P., Wang D., Cao J., Jiang T. (2024). Optimization of EEG-EMG Fusion Network for West Syndrome Seizure Detection Based on Enhanced Artificial Rabbit Algorithm. IEEE Trans. Instrum. Meas..

[31] Cui R., Chen W., Li M. (2024). Emotion recognition using cross-modal attention from EEG and facial expression. Knowl.-Based Syst..

[32] Sitnikova E., Hramov A.E., Koronovsky A.A., Van Luijtelaar G. (2009). Sleep spindles and spike–wave discharges in EEG: Their generic features, similarities and distinctions disclosed with Fourier transform and continuous wavelet analysis. J. Neurosci. Methods.

[33] Wu F., Mai W., Tang Y., Liu Q., Chen J., Guo Z. (2022). Learning spatial-spectral-temporal EEG representations with deep attentive-recurrent-convolutional neural networks for pain intensity assessment. Neuroscience.

[34] Gu A., Dao T. (2023). Mamba: Linear-time sequence modeling with selective state spaces. arXiv.

[35] Peng B., Alcaide E., Anthony Q., Albalak A., Arcadinho S., Biderman S., Cao H., Cheng X., Chung M., Grella M. (2023). Rwkv: Reinventing rnns for the transformer era. arXiv.

[36] Zhang B., Sennrich R. (2019). Root mean square layer normalization. Adv. Neural Inf. Process. Syst..

[37] Asano E., Juhász C., Shah A., Muzik O., Chugani D.C., Shah J., Sood S., Chugani H.T. (2005). Origin and propagation of epileptic spasms delineated on electrocorticography. Epilepsia.

